# ASO Author Reflections: A Symptom-Based Algorithm for Management of Granulomatous Mastitis in the United States

**DOI:** 10.1245/s10434-024-15807-7

**Published:** 2024-07-10

**Authors:** Nimmi S. Kapoor, Sarah L. Blair

**Affiliations:** 1 Department of Surgery, Division of Surgical Oncology, David Geffen School of Medicine at UCLA, Encino, Los Angeles, CA USA; 2 Department of Surgery, Division of Breast Surgery, University of California, San Diego, CA USA

## Past

Granulomatous mastitis (GM) is a benign inflammatory disease of the breast that can have a prolonged or relapsing course. Some patients will develop chronic wounds, recurrent episodes of inflammation, complex abscesses, and/or fistulas.^1,2^ Often, GM can mimic malignancy or abscess, and surgeons are frequently at the forefront of initial disease management. Once pathologic diagnosis is made, treatment regimens can vary widely, from observation alone, to surgery, immunosuppression, or multimodal treatment. Evidence-based management guidelines are limited. Most studies on GM have been conducted outside the USA, and many are retrospective chart reviews that lack information on cosmetic outcomes related to treatment.^3,4^

## Present

We sought to collect prospective data from multiple sites via an American Society of Breast Surgeons (ASBrS) registry of patients undergoing treatment for GM.^5^ Our goal was to identify treatment strategies that minimize symptom duration and achieve optimal cosmetic results, ultimately developing an algorithm to guide surgeons in managing this complex disease. We found that patients who present with most severe symptoms often undergo upfront surgery, patients who receive immunosuppression are most likely to have improvement by 1 month, and patients who receive a combination of immunosuppression and surgical intervention are more likely to have improvement and/or resolution by 1 year. Cosmesis was similar at 1 year for patients who underwent surgical intervention and/or medical intervention as long as their symptoms had improved. Intralesional steroid injection had a similar benefit to oral immunosuppression, leading to a preference for the former owing to fewer known systemic side effects. For patients with mild or moderate GM symptoms, we recommend starting treatment with intralesional steroids with or without oral nonsteroidal antiinflammatories for up to 3 months prior to starting oral immunosuppression. If symptoms persist by 1 year after starting oral immunosuppression, surgical intervention may be warranted. On the other hand, patients with more severe GM symptoms can be started on upfront oral steroids with surgical intervention for local symptoms if warranted. Persistent or relapsing symptoms may require other immune-modulating agents, addition of intralesional steroids, or further surgery by 1 year.

## Future

This United States-based ASBrS registry study provides a groundwork for clinicians to manage patients presenting with biopsy-proven granulomatous mastitis. While we found that immunosuppression, in the form of oral steroids or intralesional injections, was most likely to result in improvement of symptoms, there is still a role for surgical intervention in some cases. Although GM is uncommon, it is important for breast surgeons to be aware of a multimodal, symptom-based treatment approach. Future studies evaluating the extent of surgical intervention and most effective immunosuppression regimens will be useful to further guide clinical decision-making.
